# Extraction of total flavonoids from *Chaenomeles speciosa* (Sweet) Nakai and its antioxidant and lipoxygenase inhibition effects

**DOI:** 10.1371/journal.pone.0320582

**Published:** 2025-04-01

**Authors:** Wenqing Pei, Yuting Sun, Juan Li, Yupei Zhang, Chenkang Jian, Feng Lu, Ali Tao, Qizhao Li

**Affiliations:** 1 School of Pharmacy, Anhui Xinhua University, Hefei, China; 2 Anhui Intai Technology Co., Ltd, Hefei, China; Universidad San Francisco de Quito - Campus Cumbaya: Universidad San Francisco de Quito, ECUADOR

## Abstract

Ultrasound-assisted extraction technology was utilized to extract total flavonoids from *Chaenomeles speciosa* (Sweet) Nakai, and response surface methodology was employed to optimize the extraction process. The anti-oxidant and lipoxygenase inhibitory activities were evaluated, along with an analysis of the type of inhibition. The results revealed that the optimal extraction conditions for total flavonoids from *Chaenomeles speciosa* (Sweet) Nakai were as follows: an ethanol concentration of 62%, a liquid-to-solid ratio of 15:1 mL/g, an ultrasonic temperature of 68°C, and an ultrasonic time of 40 min, resulting in a total flavonoid extraction rate of 10.18%. Antioxidant assays demonstrated that the *Chaenomeles speciosa* (Sweet) Nakai extract exhibited significant radical scavenging activities against 1,1-diphenyl-2-picrylhydrazyl radicals, 2,2’-azinobis (3-ethylbenzothiazoline-6-sulfonic acid ammonium salt) radicals, and hydroxyl radicals, with IC_50_ values of 582 µg/mL, 538 µg/mL, and 1709 µg/mL, respectively. Furthermore, enzyme inhibition assays indicated that the *Chaenomeles speciosa* (Sweet) Nakai extract possesses notable inhibitory activity against lipoxygenase, with an IC_50_ value of 2658 µg/mL. This inhibition is mediated through a mixed reversible inhibition mechanism.

## 1. Introduction

*Chaenomeles speciosa* (Sweet) Nakai, a member of the Rosaceae family, is the dried, near-ripe fruit of the plant, commonly found in central, eastern, and south-western China, with a long history of use in traditional medicine. Scholars have identified various bioactive compounds in *Chaenomeles speciosa* (Sweet) Nakai, such as flavonoids, terpenes, and phenolic compounds [1,2], which may contribute to its anti-inflammatory, antioxidant, immunomodulatory, antimicrobial, and antitumor activities [3,4].

Reactive oxygen species are mainly produced by cellular metabolism and are constantly cleared by the antioxidant defense system. However, elevated concentrations of reactive oxygen species can disrupt this balance, damaging cellular components such as lipids, proteins, and nucleic acids, and inducing various diseases, including inflammation, diabetes, atherosclerosis, aging, and cancer [5]. While synthetic antioxidants are a significant approach to treating diseases related to oxidative stress, their potential side effects, including liver damage and carcinogenic risks, cannot be overlooked. Lipoxygenase, a class of non-heme iron dioxygenases first discovered in legumes, is a key enzyme in the biosynthesis of leukotrienes and plays a significant role in various inflammatory diseases, such as cancer, arthritis, asthma, and allergic conditions [6]. Consequently, lipoxygenase inhibitors may offer substantial medicinal benefits in the prevention of these inflammatory diseases and represent an important class of potential drugs with significant anti-inflammatory activity.

In recent years, the quest for natural, safe, and effective antioxidants and enzyme inhibitors, particularly those derived from plants, has emerged as a prominent area of research [7,8]. Among the bioactive compounds, natural flavonoids have garnered increasing attention due to their widespread distribution and notable antibacterial, anti-inflammatory, and antioxidant properties [9]. *Chaenomeles speciosa* (Sweet) Nakai is abundant in flavonoids; however, the potential value of this plant has not yet been fully realized, despite the rich plant resources available in China [10]. Existing literature primarily focuses on the antioxidant and anti-inflammatory effects of extracts from *Chaenomeles speciosa* (Sweet) Nakai, but there remains a significant gap in the investigation of its enzyme inhibitory mechanisms, particularly regarding lipoxygenase inhibition [11,12].

To maximize the utilization of this resource, the present study aimed to optimize the total flavonoid extraction process for *Chaenomeles speciosa* (Sweet) Nakai. The total flavonoid extraction rate served as the response variable, while the effects of independent variables, including the volume fraction of ethanol, liquid-solid ratio, ultrasonic temperature, and ultrasonic time, were investigated. The extraction parameters were optimized using response surface methodology. Additionally, the antioxidant activity and lipoxygenase inhibition effects of the *Chaenomeles speciosa* (Sweet) Nakai extract were systematically evaluated, and the inhibition mechanism was analyzed. This study not only establishes a foundation for the in-depth development and utilization of *Chaenomeles speciosa* (Sweet) Nakai but also offers new insights for the creation of natural and safe anti-inflammatory drugs derived from plant extracts.

## 2. Materials and methods

### 2.1. Chemicals and reagents

1,1-diphenyl-2-picrylhydrazyl (DPPH ≥ 97%), 2,2’-azinobis (3-ethylbenzothiazoline-6-sulfonic acid ammonium salt) (ABTS ≥ 98%), quercetin (purity ≥ 98.5%), rutin (purity ≥  98%) and Soybean lipoxygenase (ECl.13.11.12, 50000 units/mg) were obtained from Aladdin (China). Vitamin C (VC) was sourced from Yuanye Biotechnology (China). All chemicals and reagents used in the study were of analytical grade.

### 2.2. Plant material sourcing and preparation

The *Chaenomeles speciosa* (Sweet) Nakai medicinal materials were purchased from Beijing Tongrentang Pharmacy and identified by Associate Professor Wang Chunli from the School of Pharmacy, East China University of Science and Technology, as the dried near-mature fruit of the Rosaceae plant, *Chaenomeles speciosa* (Sweet) Nakai. The *Chaenomeles speciosa* (Sweet) Nakai was dried at 50°C until a constant weight was achieved, then ground and passed through a 60-mesh sieve for subsequent use. A total of 2.0 g of the *Chaenomeles speciosa* (Sweet) Nakai powder was weighed, and different concentrations of ethanol were added according to various liquid-to-solid ratios. The mixture was subjected to ultrasonic extraction at a power of 200 W under varying temperatures and extraction. After cooling to room temperature, the mixture was centrifuged at 4,000 rpm for 10 min to obtain the supernatant. The residual solid was extracted once more, and the supernatants were combined and concentrated to obtain the *Chaenomeles speciosa* (Sweet) Nakai total flavonoid extract.

### 2.3. Determination of total flavonoid yield

Rutin was used as a reference standard. A total of 10 mg of rutin was dissolved in 50% ethanol and diluted to a final volume of 50 mL to obtain a 0.2 mg/mL rutin standard solution. Precise volumes of 0.0, 0.5, 1.0, 1.5, 2.0, 2.5, and 3.0 mL of this standard solution were transferred into 10 mL volumetric flasks. To each flask, 0.4 mL of 5% sodium nitrite solution was added, gently mixed, and allowed to stand for 6 min. Then, 0.4 mL of 10% aluminum nitrate solution was added, mixed, and allowed to stand for another 6 min. Next, 4 mL of 4% sodium hydroxide solution was added. Finally, 50% ethanol was added to the mark, mixed, and left to stand for 15 min. The absorbance was measured at 510 nm. A standard curve was constructed with the concentration of the rutin standard solution (C) on the x-axis and the absorbance (A) on the y-axis, allowing for the fitting of a linear regression equation [13].

1 mL of the diluted sample was analyzed under the same conditions to determine the absorbance. Based on the linear regression equation, the extraction yield of total flavonoids from the *Chaenomeles speciosa* (Sweet) Nakai was calculated using the following equation 1.


$$Extractionrate\left(\%\right)=\frac{C\times V\times N}{M}\times 100$$ (1)

where C represents the mass concentration of total flavonoids from *Chaenomeles speciosa* (Sweet) Nakai in mg/mL; V is the volume of the *Chaenomeles speciosa* (Sweet) Nakai total flavonoid extract in mL; N is the dilution factor; and M is the mass of *Chaenomeles speciosa* (Sweet) Nakai powder in grams.

### 2.4. Single-factor experiment

Precisely weighed 2.0 g of *Chaenomeles speciosa* (Sweet) Nakai powder, the extraction effects of total flavonoids from *Chaenomeles speciosa* (Sweet) Nakai were investigated under the baseline conditions of a liquid-to-solid ratio of 25 mL/g, 60% ethanol volume fraction, ultrasonic temperature of 60°C, and ultrasonic time of 40 min. The influences of different ethanol concentration (40, 50, 60, 70, and 80%) on the extraction efficiency were examined. Subsequently, the impacts of various liquid-to-solid ratios (10:1, 15:1, 20:1, 25:1, and 30:1 mL/g) were assessed. The effects of different ultrasonic temperatures (40, 50, 60, 70, and 80°C) were also investigated. Lastly, the influences of different ultrasonic times (10, 20, 30, 40, and 50 min) on the extraction efficiency were evaluated [14].

### 2.5. Response surface optimization experiment

Based on the outcomes of the single-factor experiments, where the extraction rate of total flavonoids from *Chaenomeles speciosa* (Sweet) Nakai served as the response variable, a response surface optimization experiment was conducted. Four factors were considered: ethanol concentration (A), liquid-to-solid ratio (B), ultrasonic temperature (C), and ultrasonic time (D). Each factor was assigned three levels: low, medium, and high. The Design-Expert software was utilized, employing the Box-Behnken design model to design the experiments and determine the optimal extraction conditions for total flavonoids from *Chaenomeles speciosa* (Sweet) Nakai. The experimental factors and their levels are detailed in Table 1.

**Table 1:** Test factors and levels.

Level	factor			
	A/%	B/(mL/g)	C/°C	D/min
–1	50	10	60	30
0	60	15	70	40
1	70	20	80	50

### 2.6. Determination of antioxidant capacity

The antioxidant capacity of the total flavonoid extract from *Chaenomeles speciosa* (Sweet) Nakai was assessed by its ability to scavenge 1,1-diphenyl-2-picrylhydrazyl radicals, 2,2’-azinobis (3-ethylbenzothiazoline-6-sulfonic acid ammonium salt) radicals, and hydroxyl radical (•OH) radicals, using VC as a control. The scavenging rates of 1,1-diphenyl-2-picrylhydrazyl and 2,2’-azinobis (3-ethylbenzothiazoline-6-sulfonic acid ammonium salt) radicals were determined according to the method described by Doudou and colleagues [15]. The •OH radical scavenging rate was measured following the method of Chao and colleagues [16], with each experimental group conducted in triplicate.

### 2.7. Determination of anti-lipoxygenase activity

The assay was performed with minor modifications based on the reference [17]. A 10 µ L aliquot of the sample solution was added to a test tube containing 200 µ L of enzyme solution. After incubation at 30°C for 30 min, 1.5 mL of substrate was added, and the reaction continued at 30°C for 3 min. The reaction was then stopped by the addition of 5 mL of anhydrous ethanol, followed by the addition of 5 mL of distilled water and thorough mixing. The absorbance was measured at a wavelength of 234 nm and recorded as *A*_*a*_. Absorbance *A*_*b*_ was measured using 10 µ L of distilled water in place of the sample solution, following the same procedure. Absorbance *A*_*c*_ was measured using 10 µ L of the sample solution, with 200 µ L of distilled water replacing the enzyme solution, following the same procedure. Absorbance *A*_*d*_ was measured by replacing the sample solution with 10 µ L of distilled water and adding 5 mL of anhydrous ethanol before the addition of the substrate to stop the reaction, following the same procedure. Quercetin was used as a control, and each experimental group was conducted in triplicate. The inhibition rate was calculated using Equation 2.


$$Inhibitionrate\left(\%\right)=\left(1-\frac{A_{a}-A_{c}}{A_{b}-A_{d}}\right)\times 100$$ (2)

where

*A*_*a*_ =  absorbance of the sample group.

*A*_*b*_ =  absorbance of the positive control group.

*A*_*c*_ =  absorbance of the negative control group.

*A*_*d*_ =  absorbance of the blank control group

### 2.8. Measurement of lipoxygenase inhibition kinetics

The inhibition kinetics of lipoxygenase were assessed using *Chaenomeles speciosa* (Sweet) Nakai extract at varying concentrations (5, 10, and 15 mg/mL) and lipoxygenase solutions at concentrations of 0, 5, 10, and 20 U/mL, following the methodologies described in references [17,18] with slight modifications. Readings were taken at a wavelength of 234 nm, starting initially and then every 30 seconds for a total duration of 10 min. A graph was plotted with the lipoxygenase solution concentration (E) on the x-axis and the enzymatic reaction rate (V) on the y-axis. The reversibility of lipoxygenase inhibition was determined based on whether the intersection of the linearity passed through the origin.

Building upon this, with a fixed lipoxygenase concentration of 20 U/mL, the inhibitory effects of different concentrations of *Chaenomeles speciosa* (Sweet) Nakai extract (5, 10, and 15 mg/mL) were investigated under varying concentrations of linoleic acid (0.1, 0.2, 0.3, 0.4, and 0.5 mmol/L). The Lineweaver-Burk double reciprocal plot method was employed to determine the type of inhibition exhibited by *Chaenomeles speciosa* (Sweet) Nakai extract on lipoxygenase. By transforming the Michaelis-Menten equation (3), the Lineweaver-Burk double reciprocal equation (4) can be derived, as detailed below:


$$v_{0}=\frac{V_{m}\left[S\right]}{\left[S\right]+K_{m}}$$ (3)

where *v*_0_ represents the initial reaction rate of the enzyme-catalyzed reaction; *V*_*m*_ denotes the maximum initial reaction rate of the enzyme; *K*_*m*_ is the Michaelis constant; and [*S*] refers to the substrate concentration.

By taking the reciprocal of the Michaelis equation for *v*_0_ and *V*_*m*_, one can derive *V*_*m*_ and *K*_*m*_.


$$\frac{1}{v_{0}}=\frac{K_{m}}{V_{m}}\times \frac{1}{\left[S\right]}+\frac{1}{V_{m}}$$ (4)

## 3. Results

### 3.1. Rutin standard curve

**Fig 1:**
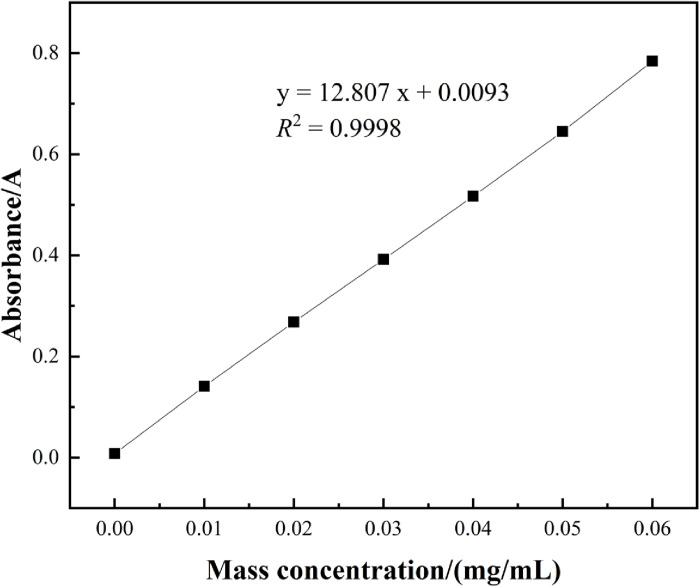
Standard curve of rutin.

The standard curve was constructed with the mass concentration of the rutin standard solution plotted on the x-axis and the absorbance on the y-axis, as illustrated in Fig 1. Within the mass concentration range of 0.00 mg/mL to 0.06 mg/mL, rutin demonstrated a strong linear relationship. The linear regression equation is given by y =  12.807x +  0.0093, with a coefficient of determination (R^2^ =  0.9998).

### 3.2. Single-factor experiments

#### 3.2.1. Effect of ethanol concentration on the extraction of flavonoids.

**Fig 2:**
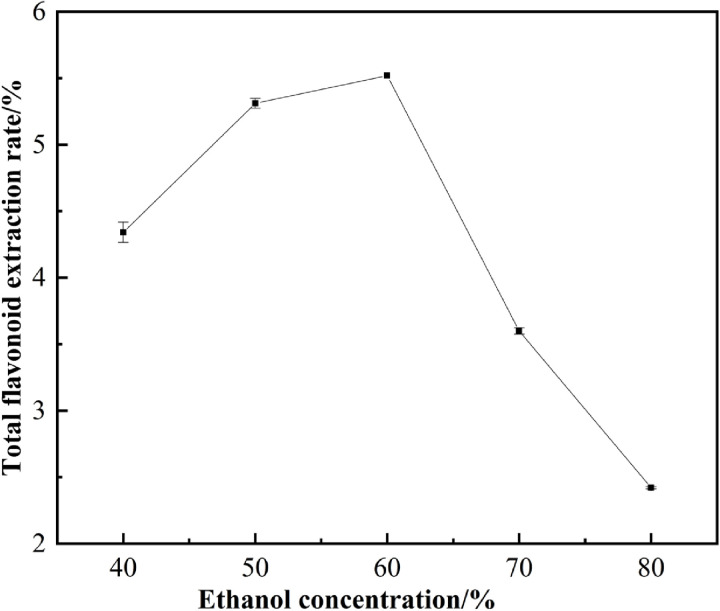
Effect of ethanol concentration on extraction of total flavonoids.

Typically, water is not the preferred solvent for extracting flavonoid compounds; however, studies have shown that adding a small amount of water to the extraction solvent can enhance the yield of the target compounds [19,20]. Consequently, a mixture of ethanol and water was selected as the extraction solvent. As illustrated in Fig 2, the extraction of flavonoids from *Chaenomeles speciosa* (Sweet) Nakai is significantly influenced by the ethanol concentration. The maximum extraction rate was achieved at an ethanol concentration of 60%. When the ethanol concentration is below 60%, the extraction rate of flavonoid compounds gradually increases with rising ethanol concentration. However, when the ethanol concentration exceeds 60%, the extraction rate sharply declines as the ethanol concentration continues to increase. These results suggest that an appropriate ethanol concentration can improve the extraction efficiency of flavonoid compounds from *Chaenomeles speciosa* (Sweet) Nakai. This may be attributed to the fact that lower ethanol concentrations enhance the solubility of flavonoid compounds in the ethanol solution, while higher ethanol concentrations may adversely affect the solubility of water-soluble flavonoids [21]. At an ethanol concentration of 60%, the highest ex-traction rate was observed, aligning with the findings of Jingyao et al. [22]. Therefore, this concentration was selected for subsequent experiments.

#### 3.2.2. Effect of liquid-to-solid ratio on the extraction of flavonoids.

**Fig 3:**
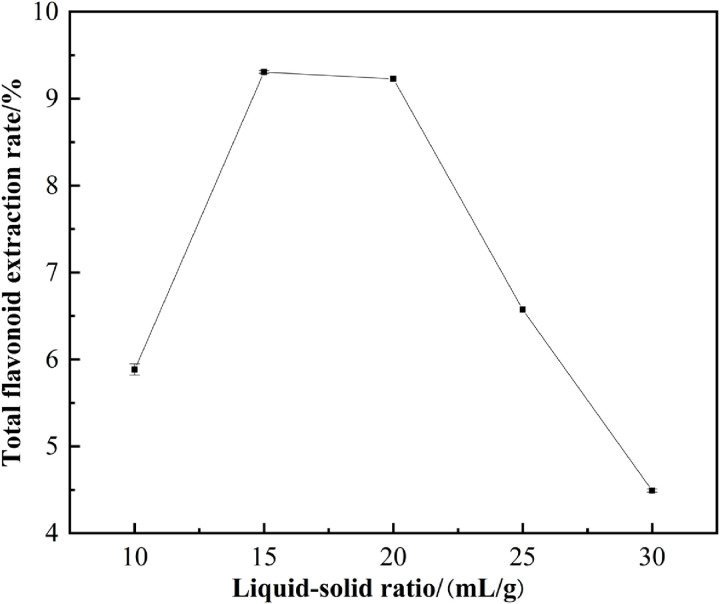
Effect of solvent to solid ratio on extraction of total flavonoids.

Variations in the liquid-to-solid ratio can affect the diffusion range of the material within the solvent, with an optimal ratio facilitating better extraction [23]. As shown in Fig 3, the extraction rate increases with the liquid-to-solid ratio from 10:1 to 15:1 mL/g; how-ever, once the ratio exceeds 15:1 mL/g, the extraction rate begins to gradually decline. This trend may be attributed to the increased contact area between flavonoids and the ethanol solvent, enhancing interactions and consequently raising the extraction rate. Nonetheless, a significantly high liquid-to-solid ratio requires more energy input, which may lead to insufficient energy for the pulverization of plant tissues and the release of bioactive com-pounds [24]. By using an appropriate amount of solvent, the extraction solvent can more effectively contact plant cells, promoting the dissolution of flavonoid compounds and in-creasing the yield of target chemicals [25]. Therefore, a liquid-to-solid ratio of 15:1 mL/g was selected for subsequent experiments, in line with the process parameters reported by Yao et al. [26] for the extraction of total flavonoids from *Cortex Lycii* and its biological activities.

#### 3.2.3. Effect of ultrasonic temperature on the extraction of flavonoids.

**Fig 4:**
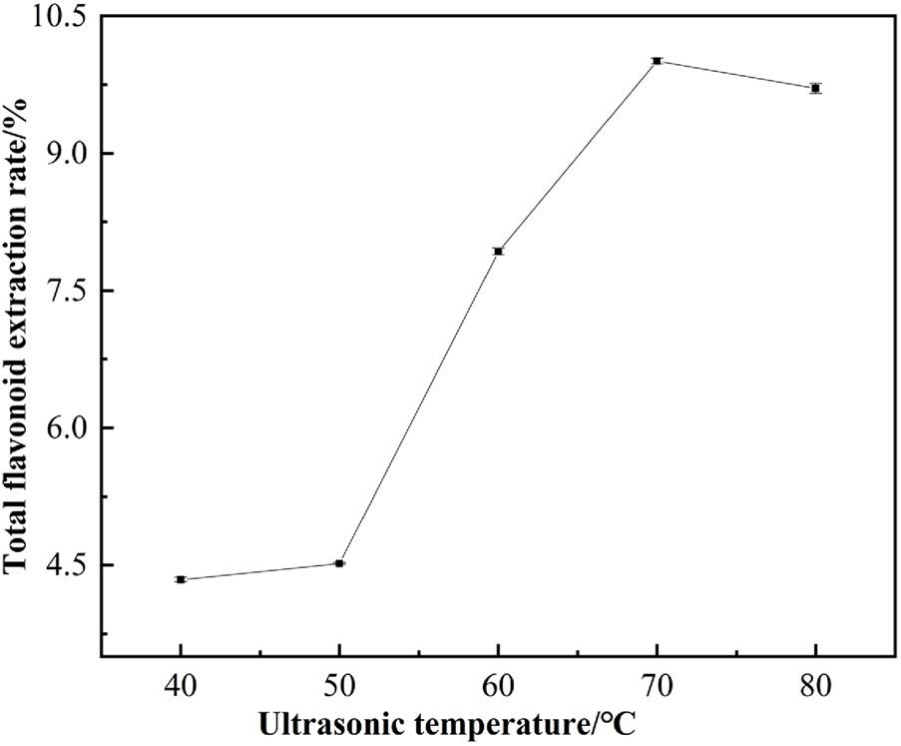
Effect of ultrasonic temperature on extraction of total flavonoids.

The enhancement of the extraction rate can be attributed to the optimal increase in extraction temperature, which facilitates improved mass transfer and reduces system viscosity [27,28]. As illustrated in Fig 4, the extraction rate of total flavonoids from *Chaenomeles speciosa* (Sweet) Nakai reaches its maximum at an elevated temperature of 70°C, after which it begins to decline. This phenomenon may be explained by the fact that increased temperature reduces solvent viscosity and enhances the rate of mass transfer. However, excessively high temperatures can lead to the degradation of thermolabile substances, resulting in a decrease in the extraction rate [29], consistent with the findings of Chang-Liang et al. [30]. These observations suggest that the target flavonoid compounds may not exhibit high thermal stability, and elevated temperatures could disrupt their structure and diminish their content. Therefore, a temperature of 70°Cwas selected for further experiments.

#### 3.2.4. Effect of l ultrasonic time on the extraction of flavonoids.

**Fig 5:**
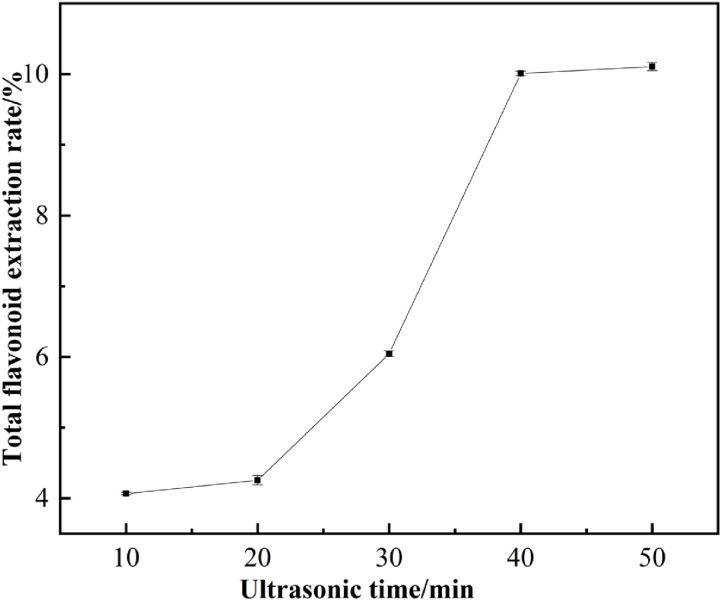
Effect of ultrasonic time on extraction of total flavonoids.

Enhancing the extraction rate also requires careful consideration of the extraction duration. The goal is typically to achieve a higher extraction rate within the shortest possible time frame. As illustrated in Fig 5, the extraction rate increases with the extension of extraction time. A rapid increase in the extraction rate is observed when the extraction time is extended from 10 min to 40 min; however, beyond 40 min, the rate of increase becomes less pronounced. This phenomenon may be related to the ultrasonic cavitation effect, where cavitation rapidly disrupts plant cell walls, releasing more flavonoids into the solvent and facilitating thorough contact between the solute and solvent, thereby enhancing the extraction rate. However, if the duration is excessively prolonged, the ultrasonic temperature may rise, potentially leading to the saturation of flavonoid solubility. Numerous studies have indicated that extending the extraction time can result in elevated temperatures, which may further contribute to the degradation and loss of bioactive compounds, as well as increased energy consumption [31]. Consequently, a duration of 40 min was selected for subsequent experiments, aligning with the research findings of An et al. [32].

### 3.3. Response surface experiments

#### 3.3.1. Results of the response surface experiments.

Based on the results of the previously described single-factor experiments, the extraction rate of total flavonoids from *Chaenomeles speciosa* (Sweet) Nakai is significantly influenced by four main parameters: ethanol concentration (A), liquid-to-solid ratio (B), ultrasonic temperature (C), and ultrasonic time (D). Consequently, 29 experiments were randomly conducted using the Box-Behnken design. Table 1 describes the four main independent variables of the Box-Behnken design and their corresponding coded levels, while Table 2 details the results of the response surface experiment. Equation (5) includes the regression equations that have been fitted to each factor by response surface analysis:

**Table 2:** Response surface test results.

Trial number	A	B	C	D	Total flavonoid extraction rate/%
1	0.000	–1.000	1.000	0.000	9.68
2	0.000	1.000	0.000	1.000	9.70
3	1.000	–1.000	0.000	0.000	9.46
4	0.000	–1.000	0.000	1.000	9.63
5	0.000	0.000	0.000	0.000	10.01
6	0.000	0.000	0.000	0.000	10.05
7	–1.000	–1.000	0.000	0.000	9.38
8	0.000	–1.000	0.000	–1.000	9.37
9	0.000	1.000	–1.000	0.000	9.58
10	1.000	0.000	–1.000	0.000	9.61
11	0.000	0.000	0.000	0.000	10.13
12	1.000	1.000	0.000	0.000	9.59
13	0.000	–1.000	–1.000	0.000	9.49
14	0.000	0.000	1.000	1.000	9.60
15	0.000	1.000	1.000	0.000	9.50
16	–1.000	0.000	1.000	0.000	9.54
17	0.000	0.000	–1.000	1.000	9.55
18	0.000	0.000	–1.000	–1.000	9.48
19	–1.000	1.000	0.000	0.000	9.39
20	0.000	0.000	0.000	0.000	10.00
21	–1.000	0.000	0.000	–1.000	9.29
22	1.000	0.000	0.000	–1.000	9.71
23	1.000	0.000	0.000	1.000	9.56
24	–1.000	0.000	–1.000	0.000	9.33
25	–1.000	0.000	0.000	1.000	9.62
26	0.000	0.000	0.000	0.000	10.03
27	0.000	0.000	1.000	–1.000	9.66
28	0.000	1.000	0.000	–1.000	9.50
29	1.000	0.000	1.000	0.000	9.51


$$\begin{array}{l} Y\;=\;10.04\;+\;0.0742A\;+\;0.0208B\;+\;0.0375C\;+\;0.0542D\;+\;0.0300AB-0.0775AC-\\ 0.1200AD-0.0675BC-0.0150BD-0.0325CD-0.3037A^{2}-0.2687B^{2}-0.2362C^{2}-\\ 0.2187D^{2} \end{array}$$ (5)

where

Y represents the extraction yield, expressed as a percentage (%).

A denotes the ethanol concentration, expressed as a percentage (%).

B signifies the liquid-to-solid ratio, measured in milliliters per gram (mL/g)

C indicates the ultrasonic temperature, measured in degrees Celsius (°C)

D indicates the ultrasonic time, measured in minutes (min)

Table 3 presents the simulated data, analysis of variance results, and regression coefficients. The model has an *F*-value of 22.92 with a *p*-value less than 0.0001, indicating the model is statistically significant. Additionally, factors A (ethanol concentration), D (ultrasonic time), AC (interaction between A and C), AD (interaction between A and D), A^2^, B^2^, C^2^, and D^2^ are all significant at the *p* <  0.05 level. The lack of fit is not significant (*p* >  0.05), with a value of 0.2982, suggesting a good fit and appropriate model prediction. The correlation coefficient R^2^ is 0.9582, and the difference between R^2^_Adj_ and R^2^_Pred_ is less than 0.2, indicating a good correlation between predicted and experimental results. Furthermore, the F-value can elucidate the degree of influence of the independent variables on the dependent variable. From Table 3, the order of influence of the four factors on the extraction rate is A (ethanol concentration) >  D (ultrasonic time) >  C (ultrasonic temperature) >  B (liquid-to-solid ratio).

**Table 3:** Results of analysis of variance.

Source	Sum of squares	Degrees of freedom	Mean square	*F*-Value	*p*-Value	Significance
Model	1.36	14	0.0969	22.92	< 0.0001	Significance
A	0.0660	1	0.0660	15.61	0.0014	Significance
B	0.0052	1	0.0052	1.23	0.2858	
C	0.0169	1	0.0169	3.99	0.0656	
D	0.0352	1	0.0352	8.33	0.0120	Significance
AB	0.0036	1	0.0036	0.8513	0.3718	
AC	0.0240	1	0.0240	5.68	0.0319	Significance
AD	0.0576	1	0.0576	13.62	0.0024	Significance
BC	0.0182	1	0.0182	4.31	0.0568	
BD	0.0009	1	0.0009	0.2128	0.6516	
CD	0.0042	1	0.0042	0.9991	0.3345	
A^2^	0.5981	1	0.5981	141.44	< 0.0001	Significance
B^2^	0.4682	1	0.4682	110.72	< 0.0001	Significance
C^2^	0.3618	1	0.3618	85.55	< 0.0001	Significance
D^2^	0.3102	1	0.3102	73.34	< 0.0001	Significance
Residual	0.0592	14	0.0042			
Lack of Fit	0.0485	10	0.0048	1.81	0.2982	Non-significance
Pure Error	0.0107	4	0.0027			
Total	1.42	28				
R^2^ = 0.9582 R^2^_Adj_ = 0.9164 R^2^_Pred_ = 0.7910						

**Fig 6:**
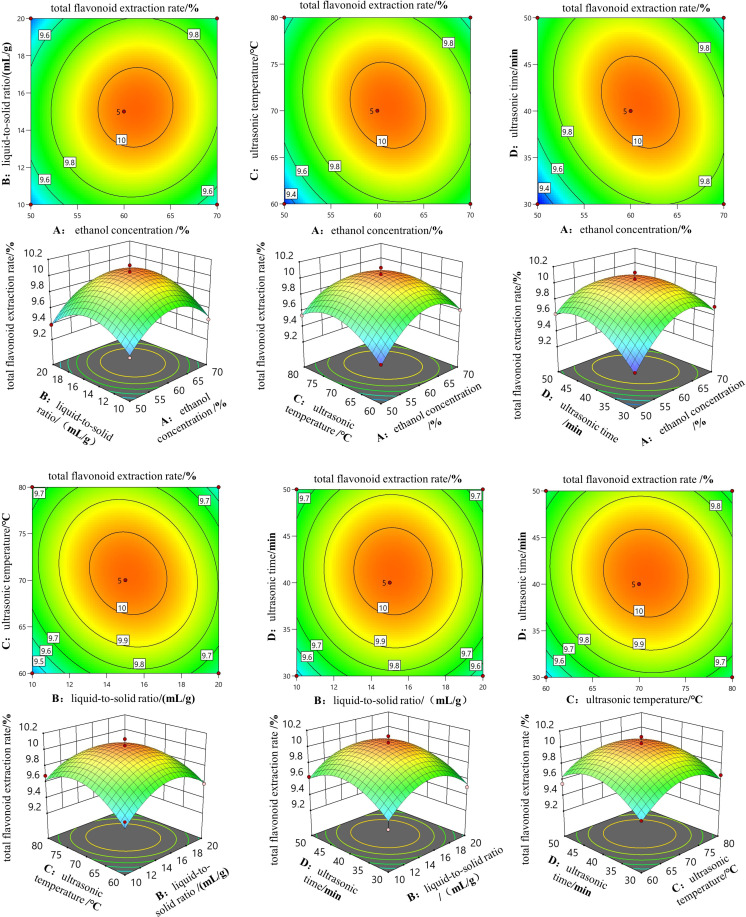
3D response surface and contour plots.

Fig 6 reflects the impact of the four factors on the total flavonoid extraction rate. In the 3D response surface plot, steeper surfaces and elliptical contours in the contour plot indicate significant interactions. It can be observed from Fig 6 that the total flavonoid extraction rate generally follows an increasing and then decreasing trend with the in-crease of different factors. The interactions between ethanol concentration and liquid-to-solid ratio (AB), liquid-to-solid ratio and ultrasonic temperature (BC), liquid-to-solid ratio and ultrasonic time (BD), and ultrasonic temperature and ultrasonic time (CD) have no significant effect on the extraction rate (*p* >  0.05); whereas the interactions between ethanol concentration and ultrasonic temperature (AC), and ethanol con-centration and ultrasonic time (AD) significantly affect the extraction rate (*p* <  0.05), consistent with the analysis of variance results.

The model generated conditions for ultrasonic-assisted extraction based on the response surface methodology, optimized for the active flavonoid compounds in *Chaenomeles speciosa* (Sweet) Nakai: an ethanol concentration of 62.21%, a liquid-to-solid ratio of 15.14 mL/g, an ultrasonic temperature of 67.65°C, and an ultrasonic time of 40.05 min, with a simulated value of 10.03%. Considering the current situation, the operational conditions were finally adjust-ed as follows: an ethanol volume fraction of 62%, a liquid-to-solid ratio of 15 mL/g, an ultrasonic temperature of 68°C, and an ultrasonic time of 40 min. A validation experiment conducted under these conditions achieved a total flavonoid extraction rate of 10.18%, which is essentially close to the predicted value. The results demonstrate that the optimization method based on the response surface methodology can accurately optimize experimental conditions and effectively predict experimental outcomes.

### 3.4. Determination of antioxidant capacity

**Fig 7:**
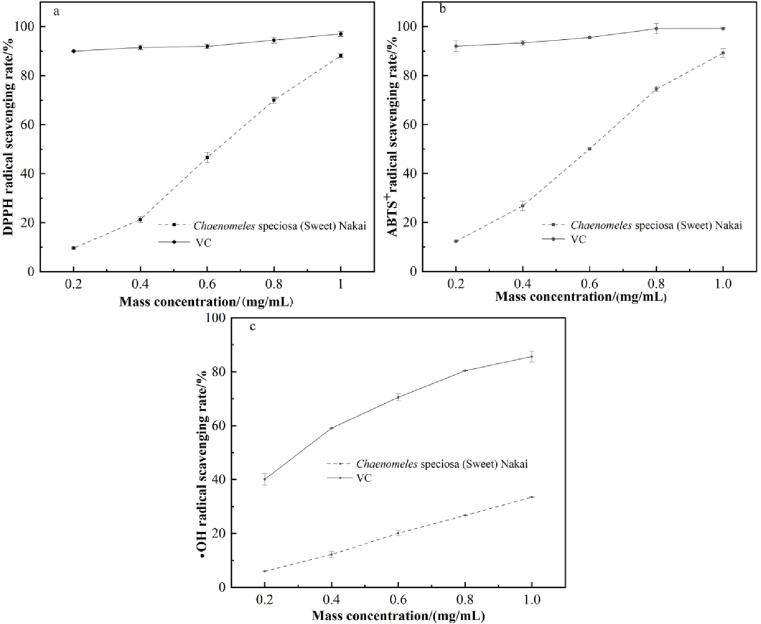
Free radical scavenging activities of *Chaenomeles speciosa* (Sweet) Nakai.

Free radicals are molecules or atoms that carry one or more unpaired electrons, enabling them to exist independently. Common examples of free radicals include hydroxyl radicals, superoxide anion radicals, and lipid peroxides [21]. Reactive oxygen species is a general term for a series of highly reactive oxygen-containing molecules and free radicals, which play a variety of roles in living organisms [33]. Reactive oxygen species include, but are not limited to, superoxide anions, hydrogen peroxide, hydroxyl radicals, monoclinic oxygen, and hypochlorous acid [34]. The antioxidant activity of the sample was assessed using the 1,1-diphenyl-2-picrylhydrazyl radical, 2,2’-azinobis (3-ethylbenzothiazoline-6-sulfonic acid ammonium salt) radical, and •OH radical methods, with the results depicted in Fig 7. It was observed that as the concentration increased, the free radical scavenging rate also rose, indicating a concentration-dependent free radical scavenging activity of *Chaenomeles speciosa* (Sweet) Nakai.

Compared to the IC_50_ values of VC against 1,1-diphenyl-2-picrylhydrazyl radicals, 2,2’-azinobis (3-ethylbenzothiazoline-6-sulfonic acid ammonium salt) radicals, and •OH radicals, which are 5 µg/mL, 28 µg/mL, and 286 µg/mL respectively, *Chaenomeles speciosa* (Sweet) Nakai exhibited superior antioxidant activity against these radicals, particularly showing strong activity in scavenging 1,1-diphenyl-2-picrylhydrazyl radicals and 2,2’-azinobis (3-ethylbenzothiazoline-6-sulfonic acid ammonium salt) radicals, with IC_50_ values of 582 µg/mL, 538 µg/mL, and 1709 µg/mL, respectively. The sample extract contains a high content of total flavonoid, which contribute to the antioxidant activity of plant extracts. For instance, Zhou et al [35] have reported a close relationship between the content of total flavonoids and antioxidant capacity.

### 3.5. Determination of anti-lipoxygenase activity

**Fig 8:**
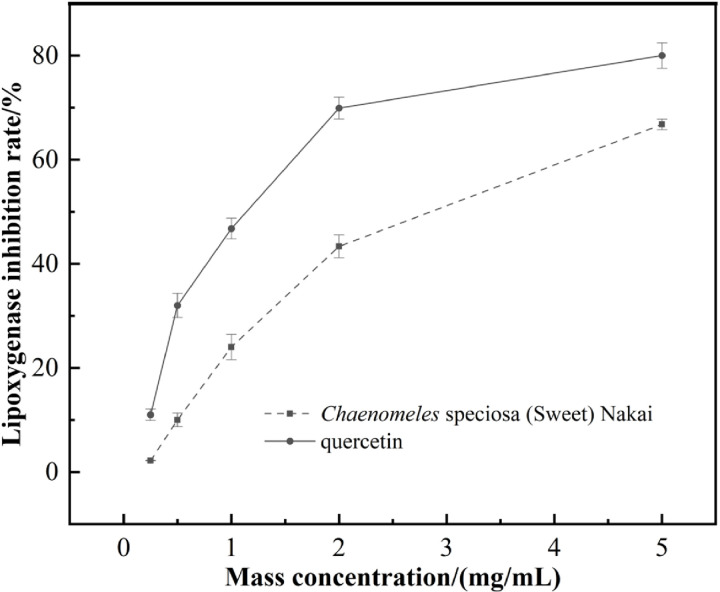
Anti-lipoxygenase of *Chaenomeles speciosa* (Sweet) Nakai.

The lipoxygenase inhibitory activity of *Chaenomeles speciosa* (Sweet) Nakai was evaluated based on its effect on the peroxidation reaction of linoleic acid. *Chaenomeles speciosa* (Sweet) Nakai contains a high concentration of flavonoids, which are known to play a crucial role in reducing the arachidonic acid cascade through the cyclooxygenase and lipoxygenase pathways, thereby alleviating inflammatory responses [36,37]. As illustrated in Fig 8, the inhibition effect exhibits a dose-dependent relationship, consistent with the findings of Zbigniew et al [38]. Com-pared to the IC_50_ value of quercetin, which is 1137 µg/mL, the IC_50_ value of *Chaenomeles speciosa* (Sweet) Nakai for lipoxygenase is 2658 µg/mL. This indicates that *Chaenomeles speciosa* (Sweet) Nakai possesses a certain degree of lipoxygenase inhibition capability.

### 3.6. Enzyme inhibition kinetics analysis

**Fig 9:**
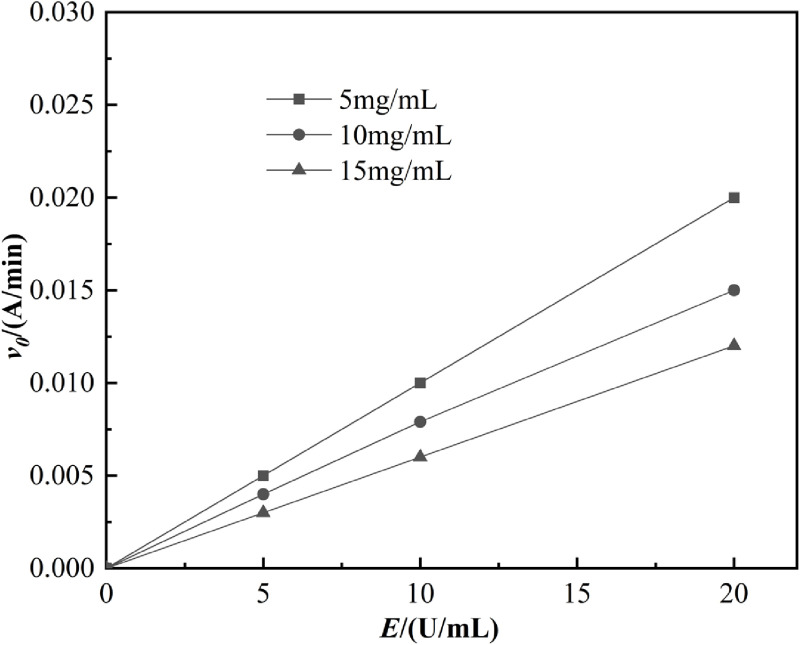
Inhibitory effect of *Chaenomeles speciosa* (Sweet) Nakai on lipoxygenase.

As shown in Fig 9, the reaction rate increases with the concentration of lipoxygenase, while it decreases with the increasing concentration of *Chaenomeles speciosa* (Sweet) Nakai extract. The slopes of the lines differ, and all pass through the origin, indicating that *Chaenomeles speciosa* (Sweet) Nakai exerts a reversible inhibitory effect on lipoxygenase. This form of inhibition suggests that the inhibitor binds to the enzyme via non-covalent interactions without undergoing a chemical reaction, and can be disrupted by physical means [39]. Several common natural plants, such as matsutake mushrooms and grapefruit, also exert their effects on corresponding active enzymes through reversible inhibition [40].

**Fig 10:**
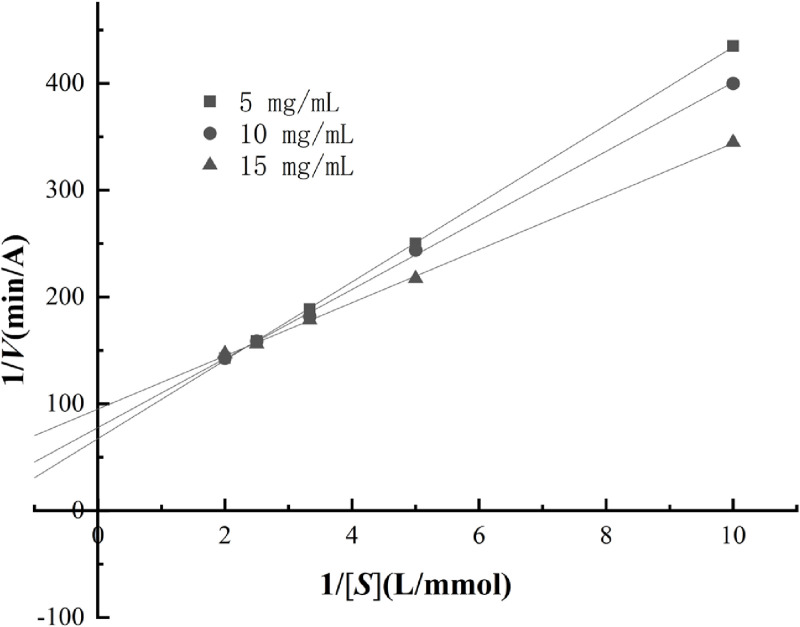
Lineweaver-Burk curve of *Chaenomeles speciosa* (Sweet) Nakai inhibition on lipoxygenase.

According to the Lineweaver-Burk plot, when the fitted line intersects the x-axis, it indicates non-competitive inhibition; when it intersects the y-axis, it indicates competitive inhibition; and when it intersects within the first quadrant, it indicates mixed inhibition [28]. As illustrated in Fig 10, the Lineweaver-Burk plot for *Chaenomeles speciosa* (Sweet) Nakai’s inhibition of lipoxygenase intersects in the first quadrant. Furthermore, with increasing concentrations of *Chaenomeles speciosa* (Sweet) Nakai extract, both *K*_*m*_ and *V*_*m*_ decrease, suggesting that the type of inhibition exhibited by *Chaenomeles speciosa* (Sweet) Nakai on lipoxygenase aligns with a mixed inhibition pattern.

## 4. Conclusions

The results indicated that *Chaenomeles speciosa* (Sweet) Nakai is a plant resource with a high total flavonoid content. Response surface methodology was employed to optimize the ultrasonic extraction process, and the optimal extraction conditions were established: an ethanol concentration of 62%, a liquid-to-solid ratio of 15:1 mL/g, an ultrasonic temperature of 68°C, and an ultrasonic duration of 40 min. Under these conditions, the total flavonoid extraction rate reached 10.18%. The results of the antioxidant assay demonstrated that the extract of *Chaenomeles speciosa* (Sweet) Nakai exhibited significant radical scavenging activities against 1,1-diphenyl-2-picrylhydrazyl radicals, 2,2’-azinobis (3-ethylbenzothiazoline-6-sulfonic acid ammonium salt) radicals, and hydroxyl radicals, with IC_50_ values of 582 µg/mL, 538 µg/mL, and 1709 µg/mL, respectively. Furthermore, the enzyme inhibition assay revealed that the extract of *Chaenomeles speciosa* (Sweet) Nakai displayed significant inhibitory activity against lipoxygenase, with an IC_50_ value of 2658 µg/mL. This inhibition was mediated through a mixed reversible inhibition mechanism.

The results of the study underscore the potential of *Chaenomeles speciosa* (Sweet) Nakai in the development of anti-inflammatory drugs. However, this study has certain limitations; specifically, only the total flavonoid constituents were investigated, and the specific active components were not isolated or characterized. Additionally, the bioactivity tests were primarily conducted in vitro, necessitating further validation of the effects and safety of the extracts in vivo in future research. The next phase of the study will concentrate on isolating and characterizing the active compounds, as well as exploring their specific molecular mechanisms. If the active ingredients can be successfully identified or functional extracts can be developed, these compounds may serve as promising candidates for natural anti-inflammatory agents and provide a foundation for further chemical modifications and lead drug development.

## Supporting information

S1 Table Data information for figure illustration. (XLSX)
